# Effects of traffic noise on the calling behavior of two Neotropical hylid frogs

**DOI:** 10.1371/journal.pone.0183342

**Published:** 2017-08-30

**Authors:** Valentina Zaffaroni Caorsi, Camila Both, Sonia Cechin, Rógger Antunes, Márcio Borges-Martins

**Affiliations:** 1 Programa de Pós–Graduação em Biologia Animal, Departamento de Zoologia, Instituto de Biociências, Universidade Federal do Rio Grande do Sul, Porto Alegre, Rio Grande do Sul, Brazil; 2 Programa de Pós-Graduação em Biodiversidade Animal, Universidade Federal de Santa Maria, Santa Maria, Rio Grande do Sul, Brazil; Universitat Trier, GERMANY

## Abstract

Anthropogenic disturbance has been pointed to as one of the major causes of the world´s biodiversity crisis. Among them, noise pollution is a potential underestimated threat, projected to increase in the next decades accompanying urban expansion. Rising levels of noise pollution may result in negative impacts on species highly dependent on acoustic communication. Amphibians have long served as model organisms for investigating animal acoustic communication because their reproduction depends on transmitting and receiving acoustic signals. A few studies have investigated the effects of anthropogenic noise on anurans, but there is still limited knowledge on how it affects them. In this study, we test the effect of two intensities of traffic noise on calling males of two Neotropical treefrogs species. We expect to record more changes in call parameters, to avoid masking effect, at higher intensity noise treatments, and in the species with higher call/noise frequency overlap. We performed a set of field playback experiments exposing male frogs to road noise at two different intensities (65dB and 75dB). Focal species are *Boana bischoffi* (high call/noise frequency overlap) and *B*. *leptolineata* (low call/noise frequency overlap). Both species changed acoustic parameters during or after the exposure to traffic noise. Advertisement call rate of *B*. *bischoffi* decreased during road noise, and dominant frequency decreased over time. Call length of *B*. *leptolineata* increased or decreased, depending on the order of noise intensity. We also observed spatial displacement in both species, which moved away from the noise source. Our results provide evidence that traffic noise affects anuran calling behavior, and noise intensity is an important factor affecting how species respond.

## Introduction

Habitat fragmentation, introduction of exotic species and overexploitation are among the major causes of the world´s biodiversity crisis [1]. Nevertheless, many other anthropic activities play an important role in the process of biodiversity loss. Some, however, are underestimated because their effects are more difficult to measure, especially when affecting species at a sub lethal level. Such is the case of noise pollution. Noise produced by human activities is projected to significantly increase in the next decades, accompanying urban expansion and its necessary connections, roads [2]. Rising levels of noise disturbance become a potential threat for many species, especially those depending on transmission and detection of acoustic signals [3], because background noise may limit the distance over which animals are able to communicate [4].

A recently published review of the effects of acoustic disturbance on animals shows how immediate effects on individuals have an impact, risking species conservation [5]. Anthropogenic acoustic disturbance is affecting a wide range of animal groups, including insects [6], fishes [7,8], birds, [9–11], amphibians [12–14], and terrestrial and marine mammals [15–17]. Several species, when facing spectral overlap from background noise, display a variety of mechanisms in order to reduce masking effects, like change duration, intensities or even frequencies of their calls, even though these strategies are not always sufficient to ensure transmission and detection of signal, or subsequent mating success [5,13,18].

Amphibians are the most endangered class of vertebrates, with 42% of the extant species classified among one of the three IUCN categories of high extinction risk [19]. As anuran reproduction depends directly on emitting and perceiving sounds, if background noise interferes, limits or inhibits their communication, it may have a significant negative effect on mating success [4,5]. Anurans present a variety of communication–related adaptations, and their morphology and physiology allows them to perceive and emit sounds within a high range of frequencies, including ultrasound and seismic vibrations [20–23]. For these reasons, frogs have long served as model organisms for investigating the mechanisms, function and evolution of animal acoustic communication [24]. Studies assessing effects of anthropogenic noise on frogs have shown that species respond using distinct strategies [13,24], including changes in both temporal and spectral parameters of their calls [24] and/or the avoidance of the noise source [25,26]. To reduce the masking effect of noise, some frogs may adjust the timing of whole calls or just some notes [27,28], change call amplitude [14] or call frequency [18,29,30].

One should expect a close relationship between the degree of frequency overlap between calls and noise and the type or intensity of call modification. Indeed, species calling at frequencies within the noise spectral range will tend to be more affected [31], and therefore are more likely to have to adjust their calls towards a reduction in temporal and spectral overlap. Changes in call pattern may also be directly related to the intensity of the noise [14,32,33], as background noise can limit the distance over which an individual can perceive acoustic signals [3]. If the intensity of the noise is related to the distance from the source to the receiver [34], we would expect that anthropogenic noise emitted at lower distances (i.e. at higher intensity) would have a higher effect on anuran communication. This variation in the efficiency of signaling is proved to have major fitness consequences for other animal groups [35].

In this sense, it is imperative to determine whether the traffic road noise affects anuran males calling behavior and how animals attempt to reduce the masking effect between their signal and the noise. Furthermore, it is poorly understood how different noise intensities affect the anuran calling behavior. We hypothesize that traffic noise influences the anuran calls, depending on the extent of frequency overlap and the intensity of the noise emitted. To test this hypothesis we performed a set of field experiments intending to measure the effects of traffic noise of different intensities on the call of two anuran species in the Atlantic forest in southern Brazil. We selected one species with call frequencies highly overlapping noise frequencies and one little overlapping. We expect to record more changes in call parameters, to avoid masking effect, at higher intensity noise treatments, and in the species with higher call/noise frequency overlap.

## Material and methods

### Study area

To observe how species react to traffic noise we choose a study site with quite minimal road traffic, a research reserve, 50 km way (off-road conditions) from the closest highway. Therefore, we could simulate the effects of traffic noise upon calls of anurans not exposed to it. Experiments were conducted at the Centro de Pesquisas e Conservação da Natureza Pró–Mata, São Francisco de Paula, Rio Grande do Sul, Brazil (29°35’S, 050°15’W), from October to December 2015 (Austral Spring).

### Focal species

**Fig 1:**
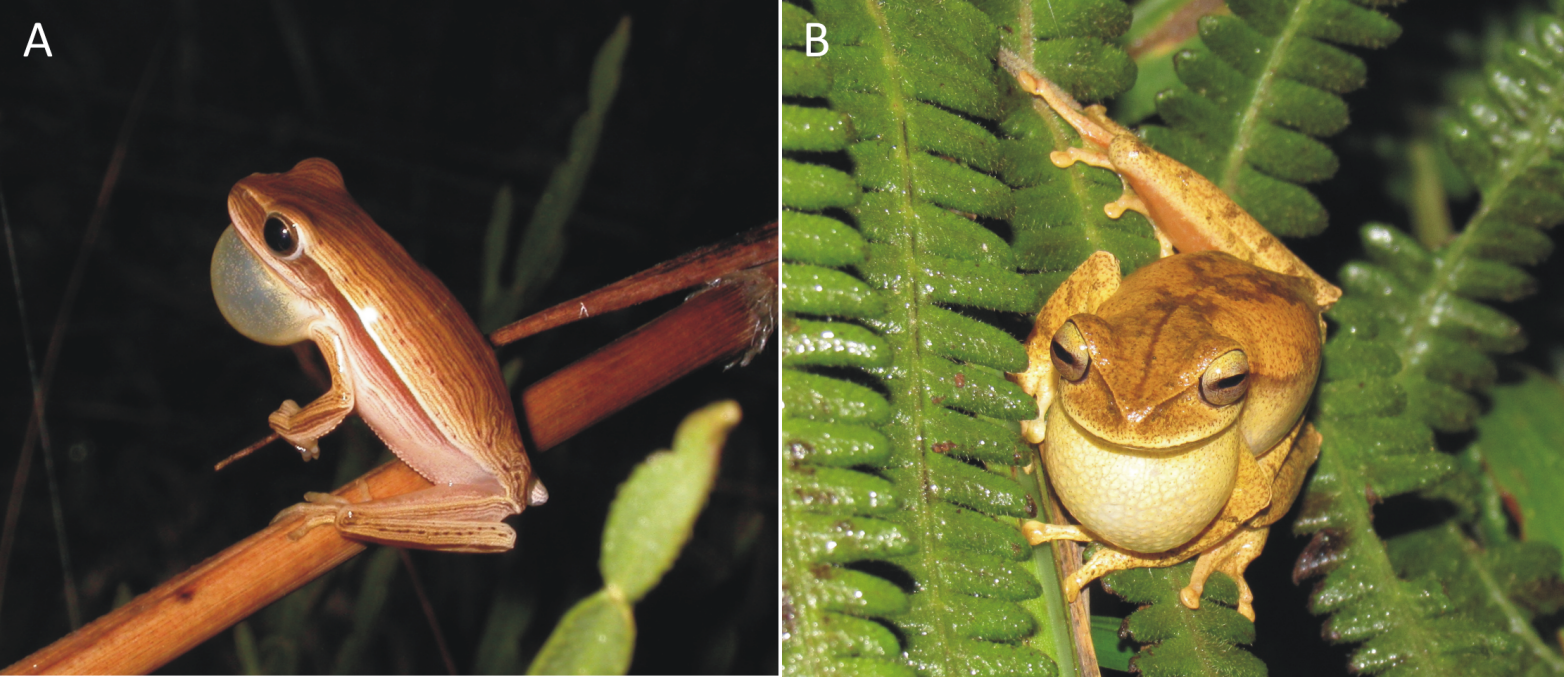
**Calling activity of (A) *Boana bischoffi* and (B) *B*. *leptolineata***.

**Fig 2:**
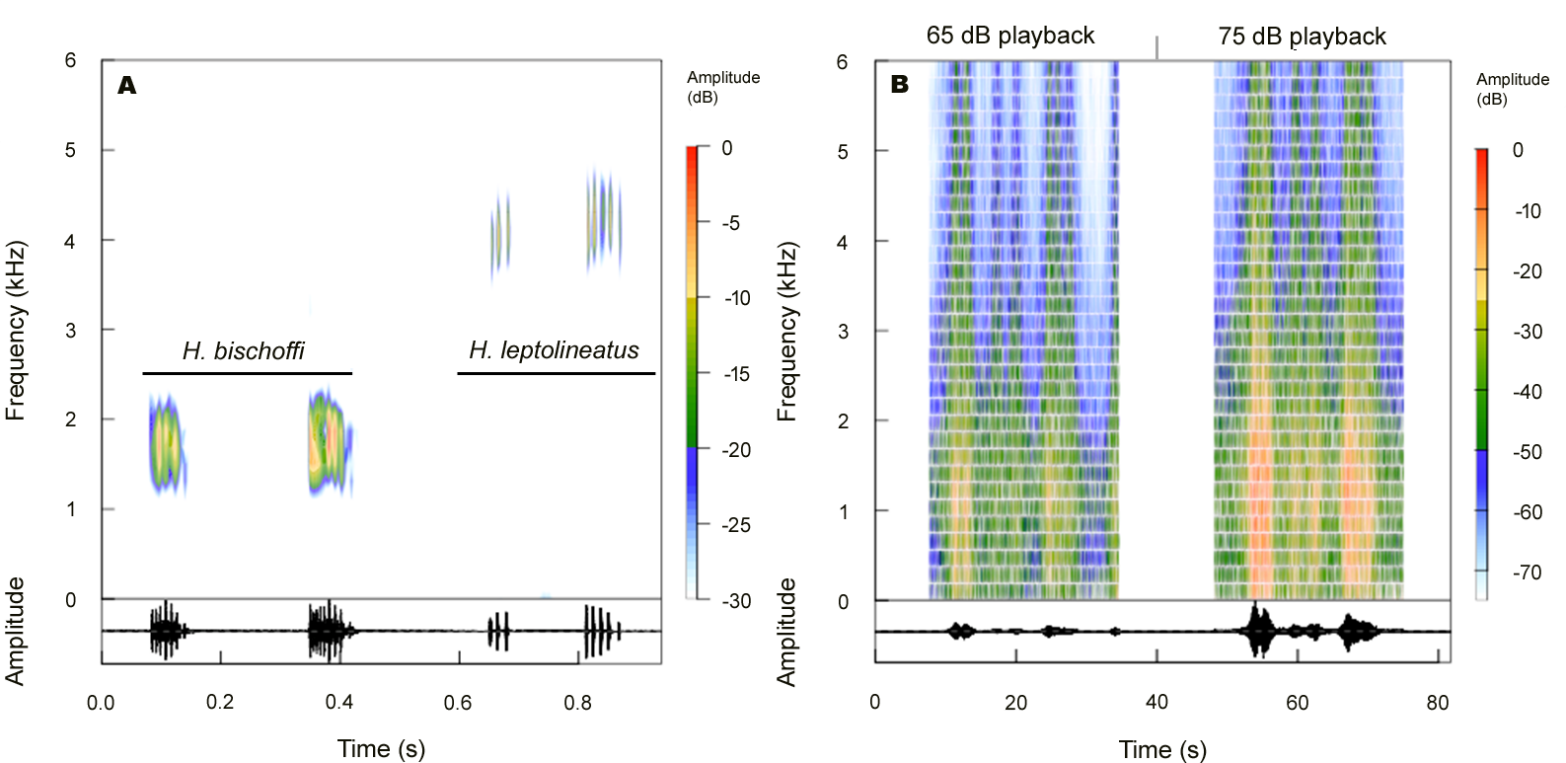
**(A) Study species advertisement calls and (B) intensities (dB) of traffic noise used on playbacks.** Spectrograms (above) and oscillograms (below) of *Boana bischoffi* and *B*. *leptolineata*.

We chose two anurans with distinct vocal profiles. The first species, *Boana bischoffi* (Fig 1A), is a medium size hylid (Snout–to–vent–length–SVL between 38–43mm), found mainly in permanent ponds close or within to forestall fragments, with two main types of call. The advertisement call is composed of one or two multipulsed notes, with duration between 0.05–0.1 seconds (Fig 2A). The call rate ranges from 3–24 notes per minute and the dominant frequency between 1.4–2.1 kHz [36,37]. The other call emitted by the species, probably territorial, is composed by one note with a series of pulses, which lasts in average 1.26s and presents dominant frequency between 1.7–2 kHz [37]. The second focal species was *Boana leptolineata* (Fig 1B), a small hylid (males SVL between 30–36mm) found mainly in open grassland on streams and ponds with clear water. It presents two main call types: i) the advertisement call of the species is composted by 3 to 4 multipulsed notes, and last from 0.04–0.1s (Fig 2A); ii) the aggressive call is longer than the advertisement call, with 11–21 pulses and lasting between 0.004–0.015s. Both calls have dominant frequency between 3.5–5.2Hz [36].

### Traffic noise

We recorded the traffic noise for the playback experiments at a major highway located in the South of Brazil (BR 389). Recordings were taken 10m from the edge of the paved road, at July 14th of 2015, beginning at 18h during winter season, for 30 minutes (S1 File). We chose this day and time for its similarity to the vehicle fluxes during the summer breeding season of the anurans in the area recorded. We used a portable sound level meter (SLM–Instrutemp ITDEC 4000, 0.1dB precision, C-weighting) to measure the mean amplitude (dB) of the traffic noise. We also measured the amplitude of the traffic noise at distances of 50m and 100m from the edge of the road. All sounds recorded in this study were obtained using a portable SONY PCM–D50 recorder, and a uni-directional microphone Sennheiser ME 67 equipped with a windscreen and a dynamic stereo headphone to monitor recordings.

### Sound editing

We used Audacity 2.1.1 software to observe and edit traffic sounds (.wav) for the playback tests. The playbacks were constructed using traffic noise and intensities previously recorded and measured on the field, as described above. The recordings used for the stimuli presented a range of frequencies from close to zero Hz up to approximately 15 kHz, with higher intensity on the lower frequencies (up to 3 kHz) and dominant frequency on 1125 Hz (dB) (Fig 2B). Sound edition included the selection of 3min traffic noise, intensity calibration (dB) for each treatment chosen and the inclusion of a silent period at the beginning and ending of each playback sound. Noise stimuli were divided into two different intensities of traffic noise: 65dB (treatment N1) and 75dB (treatment N2), which represents the mean intensity of the noise measured at 100m and 50m from the edge of the traffic road, respectively. These distances are representative to the real distances of water bodies found near roads in Rio Grande do Sul.

### Playback experiments

Playbacks followed the P1–N1–N2–P2 protocol [38] and were programed to play: three minutes of pre–stimulus (P1–silence), three of traffic noise of treatment (N1), three minutes of the treatment (N2) and for last, three minutes of post–stimulus (P2–silence), totalizing 12 minutes of playback experiment. We constructed two different playbacks ordering the treatments of traffic noise on the two possible alternative ways: Silence–65dB–75dB–Silence and Silence–75dB–65dB–Silence. Individuals were assigned to one playback type only. The first individual received the 65dB–75dB treatment and, following we alternated playback types for all others.

**Fig 3:**
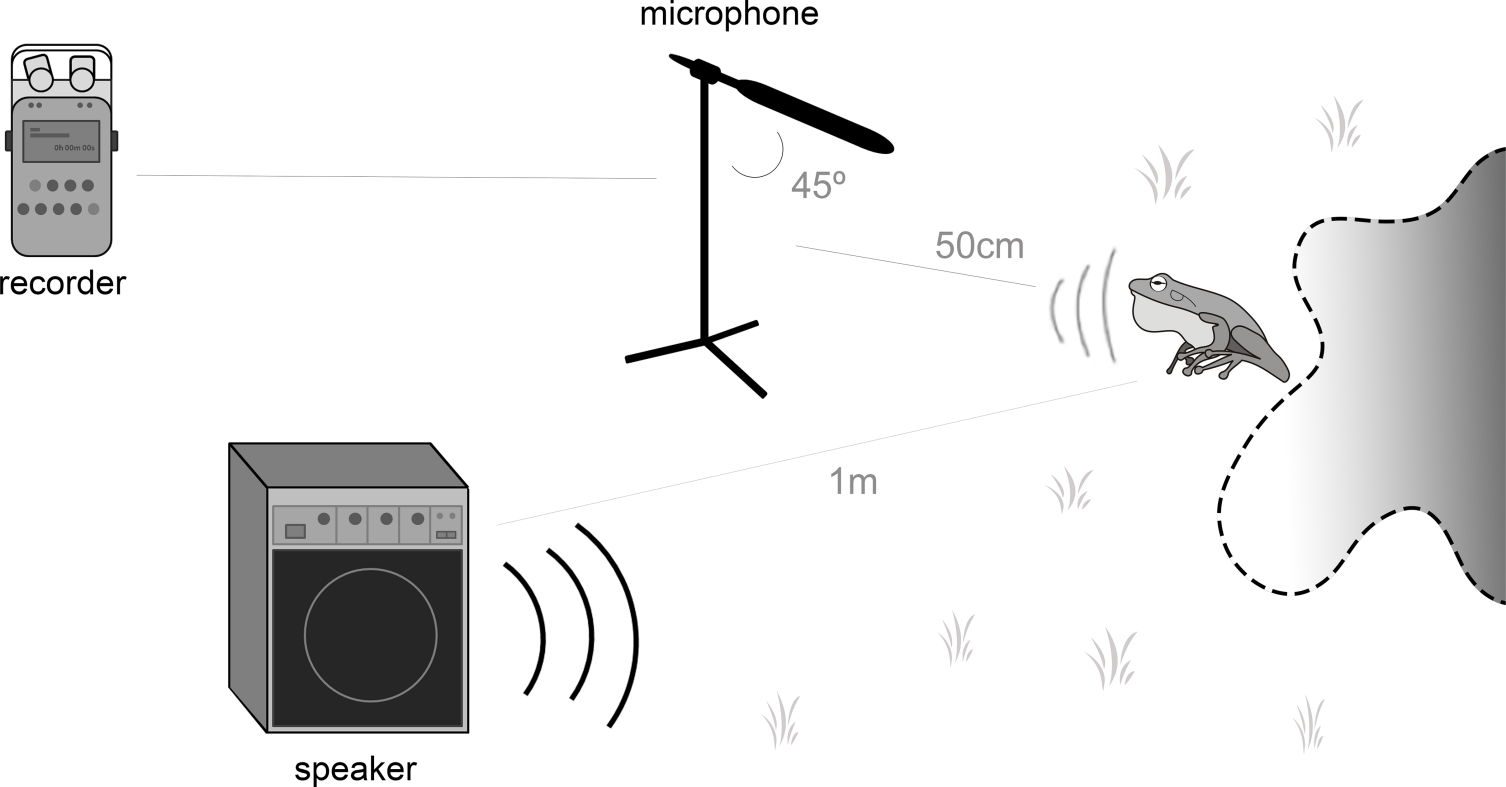
Design of experiments during the field trip to collect data on calling males.

Experiments were carried out during 18 days at dark hours, beginning one hour after the sunset until the cessation of most animals’ activity. During the study period, the air temperatures on the ponds ranged from 14.1–23.7°C, and relative humidity from 70.8–91.5%. We actively searched for calling males of the two focal species. For each individual found we implemented the following procedure: i) we actively searched for conspecific males within 5 m of the focal male and removed all those detected, to avoid any recording responses by any other individuals other than the focal male for that single experiment; ii) loudspeaker was placed at a distance of 1–4m from the animal, and the microphone within 50cm of the calling male with an inclination of 45° (Fig 3); iii) observer would get away from the focal male and waited from five to twenty minutes until the individual re-started its vocalizations; iv) playback levels were adjusted in the field using the sound level meter to reproduce the intensity observed and measured in the original road, also taking into account the distance between the focal male and the speaker; v) playbacks were performed.

The placement of the loudspeaker at different distances was necessary because its size/weight (522mm x 427mm x 267mm / 14.3Kg), which requires it to be at a stable ground. The speaker used for these experiments was carefully chosen by its characteristics to emit signals in the spectrum of frequencies of the traffic noise and do not distort low frequencies. The loudspeaker used, Oneal 360–12v, answers to frequencies from 10Hz to 70kHz and the battery lasted up to 24h on the field, so it did not need an external energy supply. After every recording, environmental sound was measured 1 m from the water body with the sound level meter.

### Specimens handling procedures, and ethical and legal permits

Once a recording was concluded, we measured male body temperature at the calling spot (using a infrared thermometer GM300, 0.1°C resolution) and hand captured it to measure body mass and SVL, using a scale to the nearest 0.1 g and a caliper (Starrett–798) to the nearest 0.1 mm respectively. Captive individuals were kept in containers for up to 5 days with vegetation and wet cotton at ambient temperatures to avoid pseudoreplication. At the end of each species experiment, the recorded individuals were released at the same water body where they were collected. All experimental procedures were approved by the applicable Brazilian biodiversity agency and local institutional committee on research and ethics: *Centro Nacional de Pesquisa e Conservação de Répteis e Anfíbios*–*Instituto Chico Mendes de Conversação da Biodiversidade* (RAN–ICMBIO–Permit No. 52021–1), by *Comissão de Pós-Graduação* (Project n° 28872—PPGBAN/UFRGS), *Comissão de Pesquisa* (COMPESQ/IB/UFRGS) and *Comissão de Ética no Uso de Animais* (CEUA/UFRGS).

### Acoustic analyses

Using Audacity 2.1.1 software, we divided each record into 3 min files, corresponding to a pre–stimulus, two stimuli and a post–stimulus time periods. Afterwards, all acoustic analyses were carried out on software Raven Pro v. 1.4 for Mac [39].

Call rate (calls– 1)/min was calculated by counting the number of calls per individual at each 3 minute interval during the playback experiment. For this parameter, we analyzed advertisement and aggressive calls separately, by counting all the signals emitted during that time period. Further, we also measured one spectral and three temporal parameters on the advertisement calls: dominant frequency (call frequency containing most energy); call length (time from the beginning to the end of one call); note length (time from the beginning to the end of one note); and interval between notes (distance between two consecutive notes) except for *Boana bischoffi* as most of the calls present a single note. These call parameters were measured by randomly selecting ten notes in *B bischoffi* and 15 notes in *B*. *leptolineata* for each 3 minute period the playback. Selection was made in Excel software (rand function; Microsoft Excel 2010. available from: https://products.office.com/pt–BR/excel). In a few cases, males emitted equal or less notes than stipulated for each species. In these cases, we used all observed notes emitted in the period to measure acoustic parameters and calculate the respective means.

### Statistical analyses

To test if noise significantly affected any of the call parameters in the two species we used a Permutational Multivariate Analysis of Variance Using Distance Matrices and post–hoc pairwise comparisons to asses which group significantly differed [40]. Stimuli type and time period (P1–N1–N2–P2) were considered as fixed factors and the individuals were considered as blocks. We also considered the order of exposure– 65/75dB or 75/65dB–as a factor. All analyses and figures were carried out in R environment [41] using Vegan: Community Ecology Package [42]; oscillograms and spectrograms were done using the Seewave package [43].

## Results

### Boana bischoffi

We recorded 19 males, and four of them showed avoidance behavior when exposed to the noise stimuli. Three individuals changed their initial position and moved away from the source of traffic noise, but remained calling. One male ceased the calling activity and apparently left the area, as we were not able to track it again. Call rates were calculated for all recorded males. Other call parameters were measured from 14 males only, due to the low quality from the recordings from a few males (moving males plus one).

**Fig 4:**
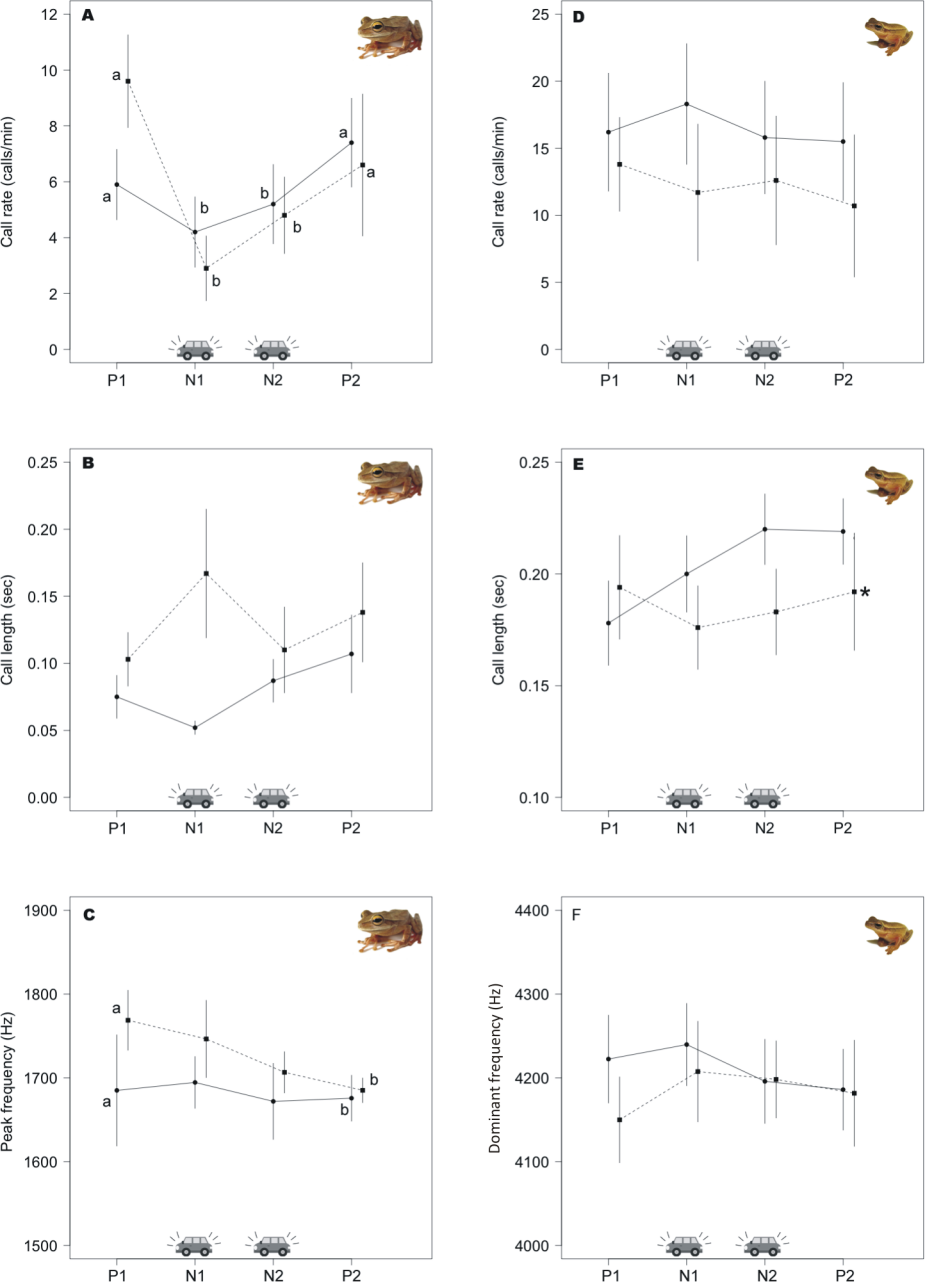
Effects of traffic noise on call parameters of the two hylids. Graphs show call parameter means (±SD) at the four periods of time inside a playback, P1 (pre–stimuli, silence), N1 (noise1), N2 (noise2), P2 (post–stimuli, silence). Dashed line represents the playback order N1 (75dB) followed by N2 (65dB) and solid line the other way around N1 (65dB) and N2 (75dB). During road noise treatments, *Boana bischoffi* decreased call rate (A). Peak frequency was significantly different for *B*. *bischoffi*, decreasing from period P1 to P2 (C). Call duration showed changes in *B*. *leptolineata* depending on the order of the treatment (E). Letters “a” and “–b” indicate statistically different values due to treatments (intensity) or playback periods, and “*” indicate differences due to playback type/order (65 or 75dB first).

Seventeen animals emitted both advertisement and aggressive calls in at least one period of the playback. Advertisement call rate was affected by traffic noise (F = 7.13; p = 0.001; Table 1), but not by time periods. The order of treatments was not significant (p > 0.05). Male calling rates significantly decreased from an average of 7.5 call/min during silence periods to an average of 4.6 and 4.3 call/min during treatments of 65 dB (F = 3.99, p = 0.012) and 75 dB (F = 3.99, p = 0.011) respectively (Fig 4A). Aggressive call rate showed no differences between stimuli types, periods or the ordination of noise (p > 0.05). Males also tended to increase the duration of their advertisement calls in response to traffic noise (Fig 4B). Advertisement calls lasted in average 0.009 sec longer in response to 75 dB traffic noise stimulus than during silence or the 65 dB stimulus although these differences were marginally non-significant (F = 1.1, p = 0.09). The order of the treatments was also marginally non-significant (F = 3.73; p = 0.06). Males first exposed to 75 dB traffic noise showed even longer calls. Males tended to change their calls to both noise intensities first presented, returning close to their original call lengths during the second noise stimuli presented. Note duration was not affected by stimulus, time period or ordination of noise (p > 0.05 for all cases). The dominant frequency differed significantly across time periods (F = 2.39; p = 0.04), decreasing from time 1 to time 4 (F = 2.07; p = 0.047) (Fig 4C). The frequency did not change in response to stimuli type, and the ordination of noise was also non-significant (p>0.05).

**Table 1:** Effects of traffic noise playback stimuli on call parameters of the focal species. Measurements of each parameter are given by means and (standard error); Dominant frequency is given in Hz. Letters “a” and “b” and numbers in bold indicate significant differences between groups.

	Time	Treatment	Aggressive call	Advertisement call				
			Call rate (call/min)	Call rate (call/min)	Call length (seconds)	Note length (seconds)	Interval (seconds)	Dominant frequency
*Boana bischoffi*	1	Silence	0.9 (0.4)	**5.9 (1.3)**^**a**^	0.07 (0.02)	0.05 (0.006)	_	**1685 (66)**^**a**^
	2	65 dB	0.5 (0.2)	**4.2 (1.3)**^**b**^	0.05 (0.005)	0.05 (0.006)	_	1694 (31)
	3	75 dB	0.9 (0.3)	**5.2 (1.4)**^**b**^	0.09 (0.02)	0.05 (0.007)	_	1672 (45)
	4	Silence	1.2 (0.4)	**7.4 (1.6)**^**a**^	0.11 (0.03)	0.05 (0.007)	_	**1676 (27)**^**b**^
	1	Silence	1.0 (0.4)	**9.6 (1.7)**^**a**^	0.10 (0.02)	0.06 (0.004)	_	**1769 (36)**^**a**^
	2	75 dB	0.5 (0.3)	**2.9 (1.6)**^**b**^	0.16 (0.05)	0.06 (0.005)	_	1746 (47)
	3	65 dB	0.7 (0.4)	**4.8 (1.4)**^**b**^	0.11 (0.03)	0.06 (0.005)	_	1706 (25)
	4	Silence	1.5 (0.8)	**6.6 (2.6)**^**a**^	0.14 (0.04)	0.06 (0.004)	_	**1685 (15)**^**b**^
*Boana leptolineata*	1	Silence	0.9 (0.3)	16.2 (4.4)	0.18 (0.02)	0.06 (0.005)	0.08 (0.005)	4222 (54)
	2	65 dB	1.2 (0.3)	18.3 (4.5)	0.20 (0.02)	0.06 (0.004)	0.08 (0.005)	4240 (49)
	3	75 dB	1.4 (0.4)	15.8 (4.2)	0.22 (0.02)	0.06 (0.004)	0.08 (0.005)	4196 (50)
	4	Silence	1.5 (0.5)	15.5 (4.4)	0.22 (0.01)^*a^	0.06 (0.005)	0.09 (0.005)	4186 (48)
	1	Silence	2.4 (0.7)	13.8 (3.5)	0.19 (0.02)	0.07 (0.007)	0.09 (0.02)	4150 (51)
	2	75 dB	1.8 (0.6)	11.7 (5.1)	0.18 (0.02)	0.07 (0.007)	0.08 (0.009)	4207 (60)
	3	65 dB	1.4 (0.7)	12.6 (4.8)	0.18 (0.02)	0.08 (0.1)	0.08 (0.01)	4198 (46)
	4	Silence	1.1 (0.7)	10.7 (5.3)	0.19 (0.03)^*b^	0.07 (0.004)	0.09 (0.01)	4182 (63)

### Boana leptolineata

We recorded 23 males. Three individuals changed their initial position to farther away of the source of noise. Nevertheless, even moving, they all continued the calling activity during playbacks. Twenty animals emitted both advertisement and aggressive call in at least one period of the noise playback. Call rates were calculated for all individuals, and other parameters for 20 males, (moving males were not used). Males did not increase advertisement call rate during the noise stimuli (Fig 4D; Table 1). Statistical analyses also did not show any significant differences between period or ordination of noise (p>0.05). Aggressive calls followed the same pattern and were not affected by stimuli type or period (p>0.05). Nevertheless, we found significant differences in advertisement call length depending on the order of noise intensity (F = 2.85, p = 0.04). Males showed progressively longer calls in response to the noise (Fig 4E), when first exposed to 65dB, and slightly shorter calls when first exposed to 75dB. Note length and dominant frequency (Fig 4F) did not change significantly in response to the period, intensity level or order (p > 0.05 for all cases).

## Discussion

In this study we found evidence that traffic noise leads to changes in anuran calls, supporting the idea that anthropogenic noise has the potential to adversely impact biodiversity [4]. Temporal parameters of the calls changed significantly during road noise treatments, affecting call rate of *Boana bischoffi* and call length *of B*. *leptolineata*. The species with low frequency call altered its dominant frequency in the last time period, after been exposed to both noises intensities for six minutes in total. Besides, we also reported a few cases of spatial displacement of males from both species, which moved away from the experimental traffic noise. Our results point out important effects of traffic noise upon frogs calling activity and shows that noise intensity is an important factor affecting species calls. Following, we discuss in detail the implications of our findings.

### Impact of traffic noise on call temporal parameters

Acoustic communication in anurans depends on the transmission and detection of signals, therefore, anthropogenic noise can have many different effects on species, especially when the interference of background noise has a masking effect on the species signaling [13]. According to this, we would expect that species whose call frequencies are within the same range of frequencies as the ones of the noise they are subject to, to present more evident changes in their acoustic behavior, potentially affecting the efficiency of their communication. Our results supported these expectations for the calling rate behavior of both species. They showed significant changes in the calling pattern of *B*. *bischoffi* during traffic noise stimuli, the species with high call/traffic noise frequency overlap. *Boana letptolineata*, with low spectral overlap, kept similar calling rates during stimuli.

Both intensities of traffic noise stimuli affected *B*. *bischoffi* call rate. It decreased more than 60% in average at both noise intensities, 65dB and 75dB, when compared to pre and post–stimulus periods (silence). These intensities represent traffic noise at 100m and 50m from the edge of the road, respectively, showing that for this species the traffic noise has a strong effect on its calling activity even at these distances. In a study with *Hyla chrysoscelis* female frogs, Bee and Swanson (2007) reported increases in latency response and decreases in orientation towards the target signal (artificial calling male) directly related with an increase in the intensity of traffic noise (37 e 85dB). Therefore, traffic noise not only leads to a decrease in call rate emission by males, but potentially results in a lower call detection efficiency by females. This may have a significant synergistic deleterious effect in mate selection, which is yet to be better investigated [34,44]. Anuran species decreasing signal rate when exposed to noise, like *B*. *bischoffi*, were observed in several species of Hylidae, Microhylidae and Ranidae to different sources of noise, either natural or anthropic, such as air plane, motorcycle engine and traffic noise [12,14,26]. Males of different species appear to recognize when their signal is more likely to be transmitted and detected, avoiding periods of maximal interference based on the total background noise (native + artificial/anthropic stimuli) of the pond.

On the other hand, we did not detect changes on call rate for *B*. *leptolineata*. This result corroborates our initial hypothesis that the species with high frequency call and little spectral overlap would be less affected by traffic noise. Such absence of response would be related to the little overlap between the signal and the background noise, as seen in other species calling in higher frequencies [14]. Still, *B*. *leptolineata* is known to change call rate in response to the calls of invasive frogs, even when their calls present little spectral overlap, as well as in response to continuous white noise [31]. In sum, these results point out that adjustments in call rate are likely to be stimulus-specific, and that the triggers for that adjustment are yet to be fully explored.

Only a few studies have tested the effect of anthropogenic noise on parameters other than call rate [28,30,45]. Our results showed that call length of *B*. *bischoffi* also tended to be affected, specially when males were exposed first to the most intense stimulus. Males slightly increased call length in this condition, although were results were only marginally significant. However, when analyzing temporal parameters of the call of *B*. *leptolineata*, we found that when exposed to traffic noise, males seems to modify the length of their calls and their strategy depend on which intensity of noise is first presented. Males showed progressively longer calls in response to the noise when first exposed to 65dB traffic stimulus. The modification observed could be an alternative adopted to increase the temporal window of the sound produced by the animal in the environment Instead of increasing call rate as other species attempted [12,27,30,45–47], they increased call duration. Contrastingly, calling males emitted slightly shorter calls after being first exposed to 75dB noise. Therefore, the noise intensity might be determinant to the call modification strategy to be adopted. In front of an intense noise, males may choose to not increase their call effort (more calls or longer calls), as *B*. *leptolineata* males actually did in response to the playback stimuli beginning with the less intense.

### Impact of traffic noise on call spectral parameters

The impact of noise on anuran call spectral parameters seems to be variable. Previous studies detected an increase in the dominant frequency of species whose calls overlap noise frequency range [14,29,48], however others reported a decrease [29,49] or no changes at all [28]. A recent meta-analysis comparing frequency shift responses of birds and anurans exposed to anthropogenic noises, found that while birds are prone to increase the frequency in response to noise, anurans would less commonly display such strategy [18]. Because anurans share acoustic environments among themselves, and other species for that matter, they have evolved towards emitting signals within high temporal and spatial ranges [21,23]. Nevertheless, it is plausible to expect them to adjust their tones and timing to workaround the masking effect problem.

*Boana bischoffi* males decreased call’s dominant frequency in response to traffic noise. This species calls at 1.7kHz, so it is not feasible an increase beyond 3kHz (frequency at which the energy of the traffic noise decreases), once frequency changes usually not exceed 300Hz in anurans [14,45]. Alternatively, it could be more efficient to reduce the frequency, ensuring longer distance dispersion of the signal [44,50]. For the high pitch call species studied, *B*. *leptolineata*, we did not detect any changes in call dominant frequency in response to period or intensity ordination, a result consistent with previous reports for other anurans with frequencies above those of noise stimuli [14,18].

### Potential effects of traffic noise on frog’s reproductive behavior

Several studies alerted for the potential of anthropogenic originated sounds to adversely impact biodiversity, however only a few studies focused on the mechanisms behind such pattern, and tested to what extension such negative effects are due to the masking effect from the noises such as traffic. For instance, urbanized surfaces and the proximity to roads may have negative impact on the density and the presence of calling males [51,52]. We reported in this study that some individuals of *B*. *bischoffi* and *B*. *leptolineata* attempted to displace away from the source of noise, and even ceased calling. This behavior was also reported for *Hyla arborea* during manipulative experiments [28]. Our study was not designed to understand if noise might directly affect habitat selection for these species; nevertheless, it indicates a promising line of investigation. Since some anuran species have restricted distribution ranges and low dispersal capacity, their ability to move to quitter sites if background noise disrupts acoustic communication is low, therefore this topic certainly deserves the scientists’ attention [53].

All these spectral and temporal parameters are very important in mate selection and localization [44,54] and the fact that many species have developed mechanism to reduce masking effects of signal does not ensure their success on mating. In this study we observed that call modifications in response to noise might be directly related to the degree of frequency overlapping between the species call and the noise. Our study is based on a short-term exposure to traffic noise, and based on individuals not previously exposed to it. Therefore, we only accessed very immediate effects caused by noise and cannot exclude the possibility of additional changes in call parameters, which might occur in a long-term exposure. Besides, we only tested males, i.e. the emitters of acoustic signals, and exogenous acoustic noise generally decreases the ability of a receiver to decode a message [55]. It is known that female frogs exposed to traffic noise might increase the time to find and decreased orientation towards males' calls [13]. Therefore, it is yet to be understood whether changes on call parameters helps on the transmission and detection of signals emitted and if it really increases chances of mating in anthropogenic noise environments. Alternatively, it is possible that habitats such as those close to roads might work as an environmental filter for low pitch species. In this scenario, given time, we should expect a spatial effect on community composition (filtered by species call frequency) in a disturbance gradient from high to low traffic noise caused by roads.

Traffic noise is not only an alteration of transmission channel characteristics; actually, it is also a health threat that could decrease animal survival [56]. From an individual perspective, changes on calling behavior to achieve communication may have individual negative consequences, as increased exposure to predator and high energy costs [23,57]. The energetic cost of calling in frogs is well recognized [57] and so the consequences of increased vocal output in response to noise, which could lead to a use of more energy reserves [27]. Therefore, although its yet to be more explored, changing call parameter can affect not only calling activity, but indirectly the animals life function and vital rates [5,34,58].

## Supporting information

S1 File Traffic noise sample. Sample (24 seconds) of traffic noise recorded for the playback experiments at a major highway (BR 389) located in southern Brazil, Xangrilá municipality, Rio Grande do Sul. Recordings were taken 10m from the edge of the paved road, at July 14th of 2015, beginning at 18h during winter season, for 30 minutes.
(MP3)

S1 Table *Boana bischoffi* call rate. Table containing original data used for the analysis of the Call rate of *Boana bischoffi*.
(TXT)

S2 Table *Boana leptolineata* call rate. Table containing original data used for the analysis of the Call rate of *Boana leptolineata*.
(TXT)

S3 Table *Boana bischoffi* advertisement call. Table containing original data used for the analysis of the temporal and spectral parameters of *Boana bischoffi*.
(TXT)

S4 Table *Boana leptolineata* advertisement call. Table containing original data used for the analysis of the temporal and spectral parameters of *Boana leptolineata*.
(TXT)
