# Correction: Effects of traffic noise on the calling behavior of two Neotropical hylid frogs

**DOI:** 10.1371/journal.pone.0197632

**Published:** 2018-05-17

**Authors:** Valentina Zaffaroni Caorsi, Camila Both, Sonia Cechin, Rógger Antunes, Márcio Borges-Martins

The images in Fig 1 are incorrectly switched. The image that appears as A should be labeled as B, and the image that appears as B should be labeled as A. The authors have provided a corrected version below.

**Fig 1:**
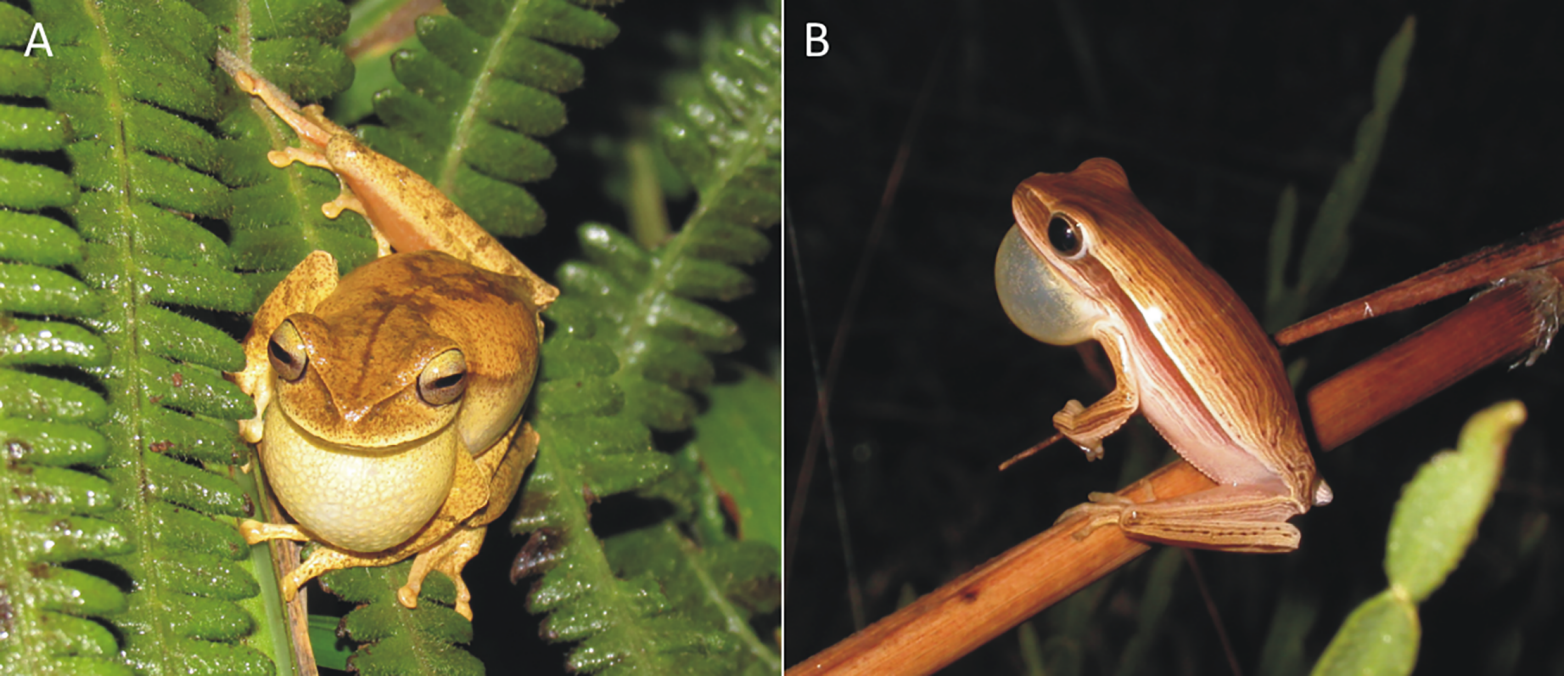
**Calling activity of (A) *Boana bischoffi* and (B) *B*. *leptolineata***.

There is an error in the caption for Fig 4, “Effects of traffic noise on call parameters of the two hylids.” Where it is written “(±SD)”, it should read “(±SE)”. Please see the corrected Fig 4 caption here.

**Fig 4:**
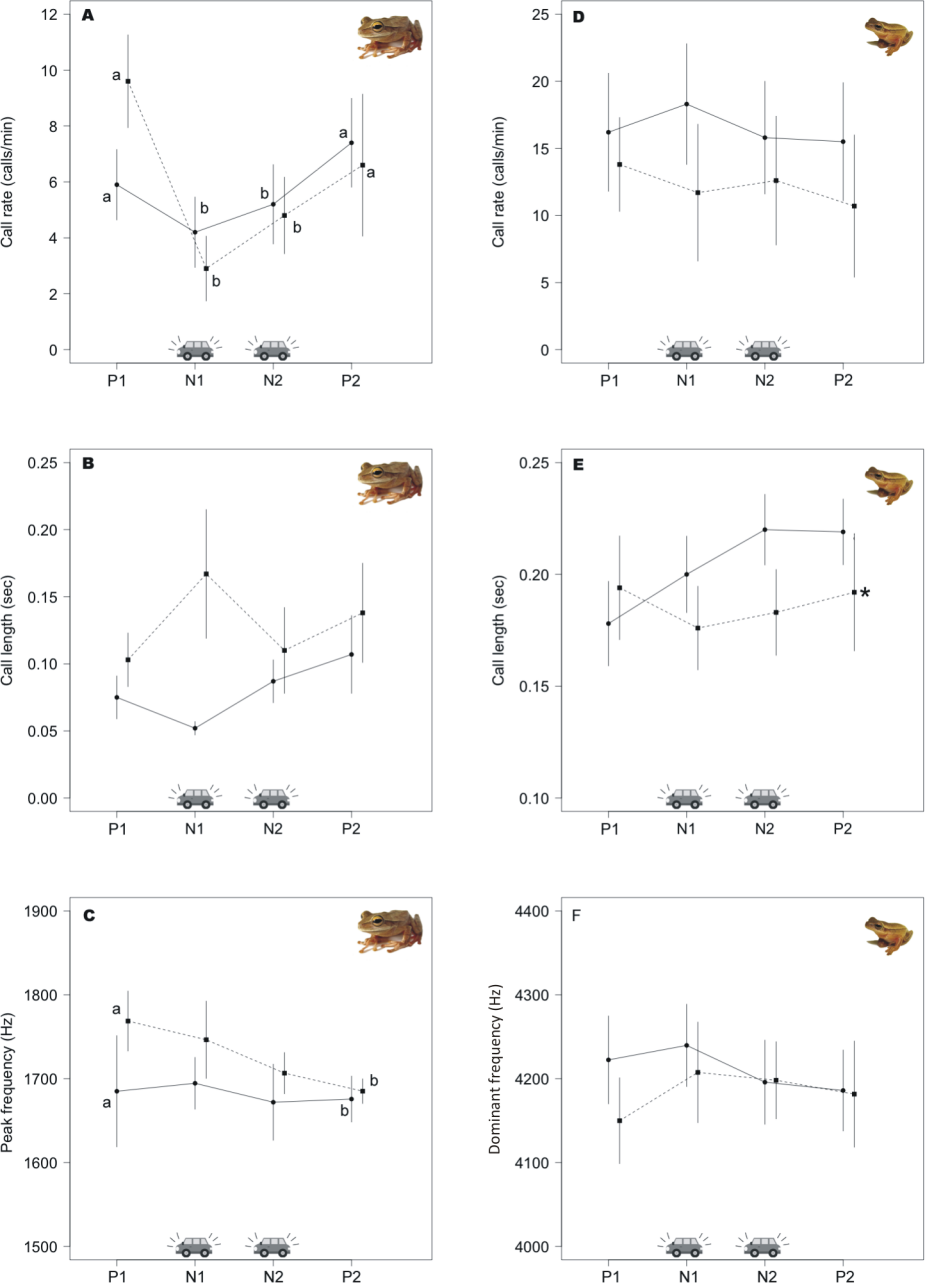
Effects of traffic noise on call parameters of the two hylids. Graphs show call parameter means (±SE) at the four periods of time inside a playback, P1 (pre–stimuli, silence), N1 (noise1), N2 (noise2), P2 (post–stimuli, silence). Dashed line represents the playback order N1 (75dB) followed by N2 (65dB) and solid line the other way around N1 (65dB) and N2 (75dB). During road noise treatments, *Boana bischoffi* decreased call rate (A). Peak frequency was significantly different for *B*. *bischoffi*, decreasing from period P1 to P2 (C). Call duration showed changes in *B*. *leptolineata* depending on the order of the treatment (E). Letters “a” and “–b” indicate statistically different values due to treatments (intensity) or playback periods, and “*” indicate differences due to playback type/order (65 or 75dB first).
